# Photorespiration plays an important role in the regulation of photosynthetic electron flow under fluctuating light in tobacco plants grown under full sunlight

**DOI:** 10.3389/fpls.2015.00621

**Published:** 2015-08-11

**Authors:** Wei Huang, Hong Hu, Shi-Bao Zhang

**Affiliations:** ^1^Key Laboratory of Economic Plants and Biotechnology, Kunming Institute of Botany, Chinese Academy of SciencesKunming, China; ^2^Yunnan Key Laboratory for Wild Plant ResourcesKunming, China

**Keywords:** CO_2_ assimilation, fluctuating light, photorespiration, photosynthetic electron flow, mesophyll conductance

## Abstract

Plants usually experience dynamic fluctuations of light intensities under natural conditions. However, the responses of mesophyll conductance, CO_2_ assimilation, and photorespiration to light fluctuation are not well understood. To address this question, we measured photosynthetic parameters of gas exchange and chlorophyll fluorescence in tobacco leaves at 2-min intervals while irradiance levels alternated between 100 and 1200 μmol photons m^−2^ s^−1^. Compared with leaves exposed to a constant light of 1200 μmol photons m^−2^ s^−1^, both stomatal and mesophyll conductances were significantly restricted in leaves treated with fluctuating light condition. Meanwhile, CO_2_ assimilation rate and electron flow devoted to RuBP carboxylation at 1200 μmol photons m^−2^ s^−1^ under fluctuating light were limited by the low chloroplast CO_2_ concentration. Analysis based on the C_3_ photosynthesis model indicated that, at 1200 μmol photons m^−2^ s^−1^ under fluctuating light, the CO_2_ assimilation rate was limited by RuBP carboxylation. Electron flow devoted to RuBP oxygenation at 1200 μmol photons m^−2^ s^−1^ under fluctuating light remained at nearly the maximum level throughout the experimental period. We conclude that fluctuating light restricts CO_2_ assimilation by decreasing both stomatal and mesophyll conductances. Under such conditions, photorespiration plays an important role in the regulation of photosynthetic electron flow.

## Introduction

In nature, plants grown in open habitats usually experience changes in light intensities because of clouds. Even on clear days, the leaves of understory plants are frequently exposed to short-term fluctuating light levels due to movements by the leaves and stems of other plants above them. To cope with fluctuating light conditions, plants must regulate their photosynthetic processes. Under constant low light, most of the absorbed light energy can be used to drive photosynthesis, even when stomatal conductance (*g*_s_) is reduced. Under constant high light, the rate of CO_2_ assimilation (*A*_n_) is maintained at an elevated level due to high *g*_s_ and mesophyll conductance (*g*_m_) (Yamori et al., 2010, 2011). Fluctuating light restricts both *g*_s_ and CO_2_ assimilation rate (Fay and Knapp, 1993; Kirschbaum et al., 1998). However, it is unclear how *g*_m_ and photorespiration respond to those changes in irradiance.

Under natural conditions, *g*_m_ is an important determinant of the CO_2_ assimilation rate, especially at high light levels (Carriquí et al., 2015). Several environmental factors, such as water status and temperature, can affect *g*_m_ (Flexas et al., 2002; Scafaro et al., 2011; Walker et al., 2013). For tobacco (*Nicotiana tabacum*) plants grown with adequate water and optimum temperature, *g*_m_ is mainly dependent upon the growth light intensity (Yamori et al., 2010). Plants exposed to strong light have higher values of *g*_m_ when compared with those grown under low light. When light levels are constant, *g*_m_ does not appear to be dependent upon light intensity (Yamori et al., 2010). However, the effect of fluctuating light condition on *g*_m_ is unclear. According to the model of Farquhar et al. (1980), CO_2_ assimilation in C_3_ plants is limited by either the carboxylation or the regeneration of ribulose-1,5-bisphosphate (RuBP). In tobacco, a model C_3_ plant, the rate of CO_2_ assimilation under high light is influenced by leaf nitrogen (N) content. CO_2_ assimilation rate under high light tends to be limited by RuBP regeneration for plants grown at high N concentration (Yamori et al., 2010, 2011). However, that presumption is based on high values of *g*_s_ and *g*_m_. Once *g*_s_ and *g*_m_ decrease because of environmental stresses such as drought, CO_2_ assimilation rate is then partially constrained by RuBP carboxylation (Flexas et al., 2002; Flexas and Medrano, 2002). Therefore, if fluctuating light levels restrict *g*_m_, then *A*_n_ likely tends to be limited by RuBP carboxylation.

Photorespiration, an inevitable process in photosynthesis, plays a supporting role in photosynthetic CO_2_ assimilation (Timm et al., 2012; Busch et al., 2013; Weber and Bauwe, 2013). This process is initiated by the oxygenation of RuBP, in which one molecule of glycolate-2-phosphate and one molecule of glycerate-3-phosphateare are produced (Ogren, 1984). Although glycolate-2-phosphate cannot be used by plants for biosynthetic reactions, and is also a potential inhibitor of chloroplast functioning (Anderson, 1971), it can be converted into glycerate-3-phosphate through the photorespiratory pathway (Leegood et al., 1995). When *g*_s_ and *g*_m_ are diminished, the decreased chloroplast CO_2_ concentration increases the specificity of Rubisco to O_2_ and then induces a rise in the rate of RuBP oxygenation (Wingler et al., 1999, 2000).

Plants avoid those detrimental effects of glycolate-2-phosphate and other photorespiratory intermediates by activating the photorespiratory pathway when chloroplast CO_2_ concentration is low. In *Arabidopsis thaliana* plants grown under low irradiance, photorespiration plays a minor role in regulating photosynthetic electron flow after exposure to short-term fluctuating light (Kono et al., 2014). The growth light intensity significantly affects the development of the photorespiratory pathway (Huang et al., 2014). For example, plants such as tobacco grown under bright light have a greater capacity than those under low light (Huang et al., 2014). However, little is known about how the photorespiratory pathway functions in the acclimation to fluctuating light by plants grown under high light. Because this pathway is critical to the control of *A*_n_ and photosynthetic electron flow (Takahashi et al., 2007; Timm et al., 2012; Huang et al., 2014), it is important that research focused on photosynthetic regulation under fluctuating light should include growth light intensity as an experimental variable.

In this study, we measured the photosynthetic parameters of gas exchange and chlorophyll fluorescence to investigate the responses of *g*_m_, *A*_n_, and photosynthetic electron flow to fluctuations in light levels. We also examined the limiting step of CO_2_ assimilation and the role the photorespiratory pathway has in modulating photosynthetic electron flow under alternating light conditions. Our objective was to improve our understanding of how photosynthesis is regulated when sun-grown plants are exposed to changes in irradiance. The following questions were addressed: (1) Is *g*_m_ restricted by fluctuating light? (2) What is the limiting step of *A*_n_ under fluctuating light? and (3) Does the photorespiratory pathway play an important role in regulating photosynthetic electron flow under fluctuating light?

## Materials and methods

### Plant materials and growing conditions

Following seed germination, seedlings of tobacco cv. y87 were cultivated in phytotron for 7 weeks. Afterwards, they were grown in plastic pots in an open field at Kunming Institute of Botany, Yunnan, China (elevation 1900 m, 102°41′E, 25°01′N). During our experiment period (10 May to 24 June 2013), none of the plants experienced any water or nutrient stresses. The average temperature at Kunming was 20.9°C in May and 20.6°C in June. Fully expanded mature leaves on 13-week-old plants were used for photosynthetic measurements.

### Analyses of gas exchange, chlorophyll fluorescence, and mesophyll conductance

Photosynthetic parameters for gas exchange and chlorophyll fluorescence were monitored with an open gas exchange system that incorporated infrared CO_2_ and water vapor analyzers (Li-6400XT; Li-Cor Biosciences, Lincoln, NE, USA) and a 2-cm^2^ measuring head (6400-40 Leaf Chamber Fluorometer; Li-Cor Biosciences). Measurements were made in a phytotron where relative air humidity (60%) and air temperature (25°C) were controlled. The atmospheric CO_2_ concentration was maintained at 400 μmol mol^−1^ by the Li-6400XT. To generate a light response curve, we initially exposed the mature leaves to strong irradiance (2000 μmol photons m^−2^ s^−1^) for 20 min to obtain steady, high levels of *g*_s_ and CO_2_ assimilation. Afterward, photosynthetic parameters were evaluated at 2-min intervals at photosynthetic photon flux densities (PPFDs) of 2000, 1600, 1200, 800, 500, 300, 200, 100, 50, 20, or 0 μmol photons m^−2^ s^−1^. To investigate the responses of *g*_s_, *g*_m_, CO_2_ assimilation rate, and photosynthetic electron flow to fluctuating light, we also evaluated those photosynthetic parameters under light levels that alternated every 2 min between 100 and 1200 μmol photons m^−2^ s^−1^ after dark-adaptation for 30 min. Photosynthetic induction curves were also developed at 1200 μmol photons m^−2^ s^−1^ after 30 min of darkness. Values for those parameters were recorded automatically by the Li-6400XT at 2-min intervals.

The CO_2_ assimilation rate vs. chloroplast CO_2_ concentration (*C*_c_) was examined at 1200 μmol photons m^−2^ s^−1^ (von Caemmerer and Farquhar, 1981). For each *A*_n_/*C*_c_ curve, the photosynthetic rate reached a steady state at 400 μmol mol^−1^ CO_2_, then decreased to a lower limit of 50 μmol mol^−1^ before increasing stepwise to an upper limit of 1600 μmol mol^−1^. Each stepwise measurement was completed within 2–3 min. Using those *A*_n_/*C*_c_ curves, we calculated the maximum rates of RuBP regeneration (*J*_max_) and RuBP carboxylation (*V*_cmax_) according to the method of Long and Bernacchi (2003).

The fluorescence parameters *F*_*o*_′, *F*_*m*_′, and *F*_*s*_ were evaluated as previously described in Baker and Rosenqvist (2004). Here, *F*_*o*_′ and *F*_*m*_′ represented the minimum and maximum fluorescence after light-adaption, respectively. *F*_*s*_ indicated the light-adapted steady-state fluorescence. The maximum quantum yield of PSII after light adaptation (*F*_*v*_′/*F*_*m*_′) was calculated as (*F*_*m*_′–*F*_*o*_′)/*F*_*m*_′. Coefficient of PSII photochemical quenching (qP) was calculated as (*F*_*m*_′–*F*_*s*_)/(*F*_*m*_′–*F*_*o*_′). Effective quantum yield of PSII (Φ_PSII_) was calculated as (*F*_*m*_′–*F*_*s*_)/*F*_*m*_′ (Genty et al., 1989).

Total photosynthetic electron flow through PSII was calculated as *J*_T_ = Φ_PSII_ × PPFD × *L*_*abs*_ × 0.5 (Krall and Edwards, 1992), where *L*_*abs*_ represented leaf absorbance and was assumed to be 0.85 for sun-grown tobacco leaves that receive high-nitrogen nutrition (Miyake et al., 2005). The constant of 0.5 was applied based on the assumption that photons were equally distributed between photosystem I (PSI) and PSII (Miyake et al., 2005). Following the assumption that the water–water cycle is not a major alternative electron sink when CO_2_ assimilation is limited (Driever and Baker, 2011), we allocated the electron flow through PSII to RuBP carboxylation (*J*_C_) and oxygenation (*J*_O_). Values for *J*_C_ and *J*_O_ were estimated according to the method of Valentini et al. (1995):

$$\begin{array}{l} J_{\text{O}}=2∕3\times \left(J_{\text{T}}-4\times \left(A_{\text{n}}+R_{\text{d}}\right)\right)\\ J_{\text{C}}=1∕3\times \left(J_{\text{T}}+8\times \left(A_{\text{n}}+R_{\text{d}}\right)\right) \end{array}$$

where *A*_n_ was the net rate of CO_2_ assimilation and *R*_d_ represented the rate of mitochondrial respiration as measured after 30 min of dark-adaptation.

We recorded values for mesophyll conductance (*g*_m_) at 1200 μmol photons m^−2^ s^−1^ after plants were exposed to either fluctuating or constant light for 60 min. For our comparisons, *g*_m_ was also estimated at 1200 μmol photons m^−2^ s^−1^ in light response curves. Values for *g*_m_ were estimated through a combination analysis of gas exchange and chlorophyll fluorescence, and according to the following equation (Harley et al., 1992; Loreto et al., 1992; Warren and Dreyer, 2006; Yamori et al., 2010, 2011):

$$\begin{array}{l} {\text{g}}_{\text{m}}=\frac{A_{\text{n}}}{C_{\text{i }}-\;\Gamma^{*}\left(J_{\text{T }}+\;8\left(A_{\text{n}}+R_{\text{d}}\right)\right)∕\left(J_{\text{T}}\;-\;4\left(A_{\text{n}}+R_{\text{d}}\right)\right)} \end{array}$$

where *A*_n_ was the net rate of CO_2_ assimilation, *C*_i_ was the intercellular CO_2_ concentration, *J*_T_ was total photosynthetic electron flow through PSII, *R*_*d*_ was the rate of mitochondrial respiration, and Γ^*^ was the CO_2_ compensation point in the absence of daytime respiration (Farquhar et al., 1980; Brooks and Farquhar, 1985), with the latter assumed to be 32.2 at 25°C (Long and Bernacchi, 2003). Using the estimated *g*_m_, we calculated the chloroplast CO_2_ concentration with the following equation (Long and Bernacchi, 2003; Warren and Dreyer, 2006; Yamori et al., 2010, 2011):

$$\begin{array}{l} C_{\text{c}}=C_{\text{i}}\;-\;\frac{A_{\text{n}}}{{\text{g}}_{\text{m}}} \end{array}$$

where *C*_*i*_ was the intercellular CO_2_ concentration, *A*_n_ was the net rate of CO_2_ assimilation, and *g*_m_ was mesophyll conductance. To identify the limiting step of CO_2_ assimilation under fluctuating light, we applied the method of Yamori et al. (2010, 2011) to determine *C*_trans_, the chloroplast CO_2_ concentration at which the transition from RuBP carboxylation to RuBP regeneration occurred:

$$\begin{array}{l} C_{\text{trans}}=\frac{K_{\text{c}}\left(1\;+\;OK_{\text{o}}\right)J_{\text{max}}∕4V_{\text{cmax }}-\;2\Gamma^{*}}{1\;-\;J_{\text{max}}∕4V_{\text{cmax}}} \end{array}$$

where *K*_c_ (μmol mol^−1^) and *K*_o_ (mmol mol^−1^) were the Michaelis constants for CO_2_ and O_2_, respectively (Farquhar et al., 1980), and were assumed to be 406.7 μmol mol^−1^ and 277 mmol mol^−1^ at 25°C, respectively (Long and Bernacchi, 2003); *J*_max_ was the maximum rate of RuBP regeneration; *V*_cmax_ was the maximum rate of RuBP carboxylation; and Γ^*^ was the CO_2_ compensation point in the absence of daytime respiration. The limiting step of CO_2_ assimilation was then determined by comparing the values of *C*_c_ and *C*_trans_.

### Statistical analysis

The results were displayed as mean values of four independent measurements. We used One-Way ANOVA and SPSS 16.0 software (SPSS Inc., Chicago, IL, USA) to examine differences among treatments involving fluctuating vs. constant light. Those differences were considered significant at *P* < 0.05.

## Results

**Figure 1:**
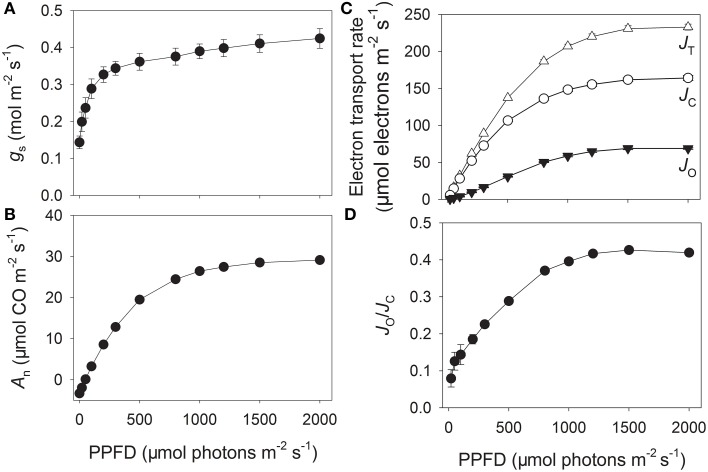
**Light response changes in stomatal conductance (*g*_s_) (A), CO_2_ assimilation (*A*_n_) (B), total electron flow through PSII (*J*_T_) (C), electron flow devoted to RuBP carboxylation (*J*_C_) (C), electron flow devoted to RuBP oxygenation (*J*_O_) (C), and *J*_O_/*J*_C_ ratio for leaves of tobacco (D)**. Measurements were conducted at 25°C and 400 μmol mol^−1^ CO_2_. Values are means ± SE (*n* = 4).

Light response curves indicated that *g*_s_ was maintained at high levels (>0.3 mol m^−2^ s^−1^) when plants were exposed to light intensities above 100 μmol photons m^−2^ s^−1^ (Figure 1A). When light levels were reduced from 100 to 0 μmol photons m^−2^ s^−1^, values for *g*_s_ decreased sharply from 0.29 to 0.14 mol m^−2^ s^−1^ within 6 min (Figure 1A). This indicated that stomatal conductance in sun-grown tobacco leaves is very sensitive to light intensity in sun-grown tobacco leaves. Under strong irradiance, i.e., 1500 μmol photons m^−2^ s^−1^, *A*_n_ was 28.5 μmol CO_2_ m^−2^ s^−1^ (Figure 1B). At levels below 1500 μmol photons m^−2^ s^−1^, values for *J*_T_, *J*_C_, *J*_O_, and *J*_O_/*J*_C_ gradually rose with increasing PPFD, peaking at 233 μmol electrons m^−2^ s^−1^, 164 μmol electrons m^−2^ s^−1^, 69 μmol electrons m^−2^ s^−1^, and 0.43, respectively (Figures 1C,D).

**Figure 2:**
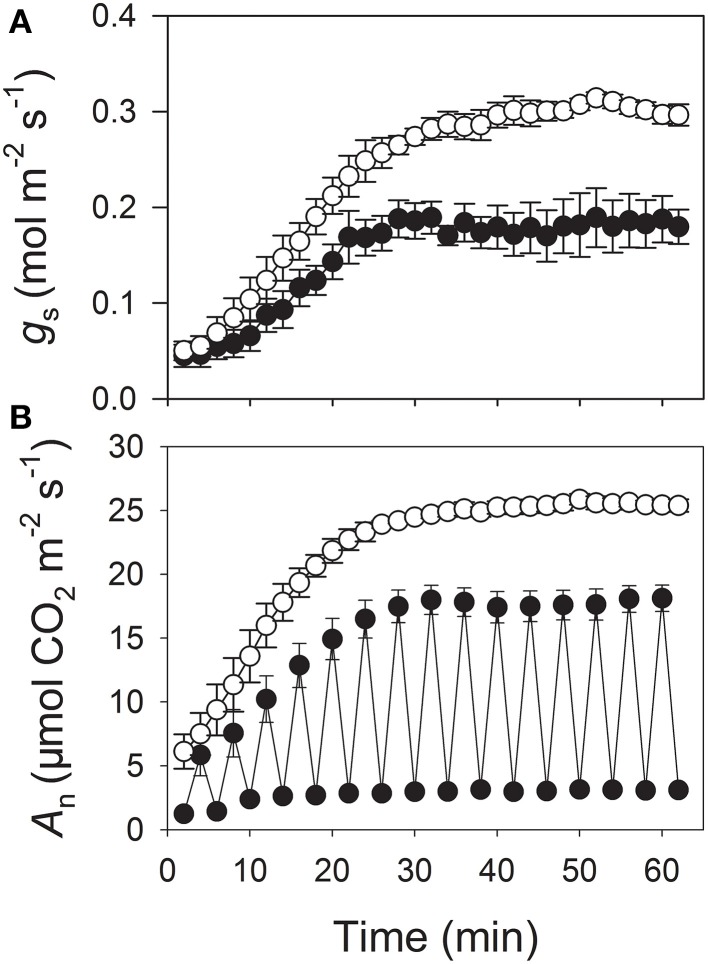
**Responses of stomatal conductance (*g*_s_) (A) and CO_2_ assimilation (*A*_n_) (B) to either light fluctuations between 100 and 1200 μmol photons m^−2^ s^−1^ at 2-min intervals (closed symbols) or constant light of 1200 μmol photons m^−2^ s^−1^ (open symbols) in tobacco leaves after 30 min of dark-adaptation**. Measurements were conducted at 25°C and 400 μmol mol^−1^ CO_2_. Values are means ± SE (*n* = 4).

Fluctuating light conditions significantly restricted the opening of stomata. After plants were alternately exposed to 100 and 1200 μmol photons m^−2^ s^−1^ every 2 min for 60 min, *g*_s_ was 0.18 mol m^−2^ s^−1^ (Figure 2A). However, when plants were illuminated at a constant 1200 μmol photons m^−2^ s^−1^ for 60 min, *g*_s_ was 0.30 mol m^−2^ s^−1^. After 60 min of fluctuating light, the CO_2_ assimilation rate at 1200 μmol photons m^−2^ s^−1^ was 18.1 μmol CO_2_ m^−2^ s^−1^ vs. 25.4 μmol CO_2_ m^−2^ s^−1^ after exposure to 1200 μmol photons m^−2^ s^−1^ for 60 min (Figure 2B). Those values for *g*_s_ and *A*_n_ differed significantly between constant and fluctuating-light treatments, demonstrating that the latter condition inhibited *g*_s_ as well as CO_2_ assimilation. This finding was consistent with those reported previously (Fay and Knapp, 1993; Kirschbaum et al., 1998).

**Figure 3:**
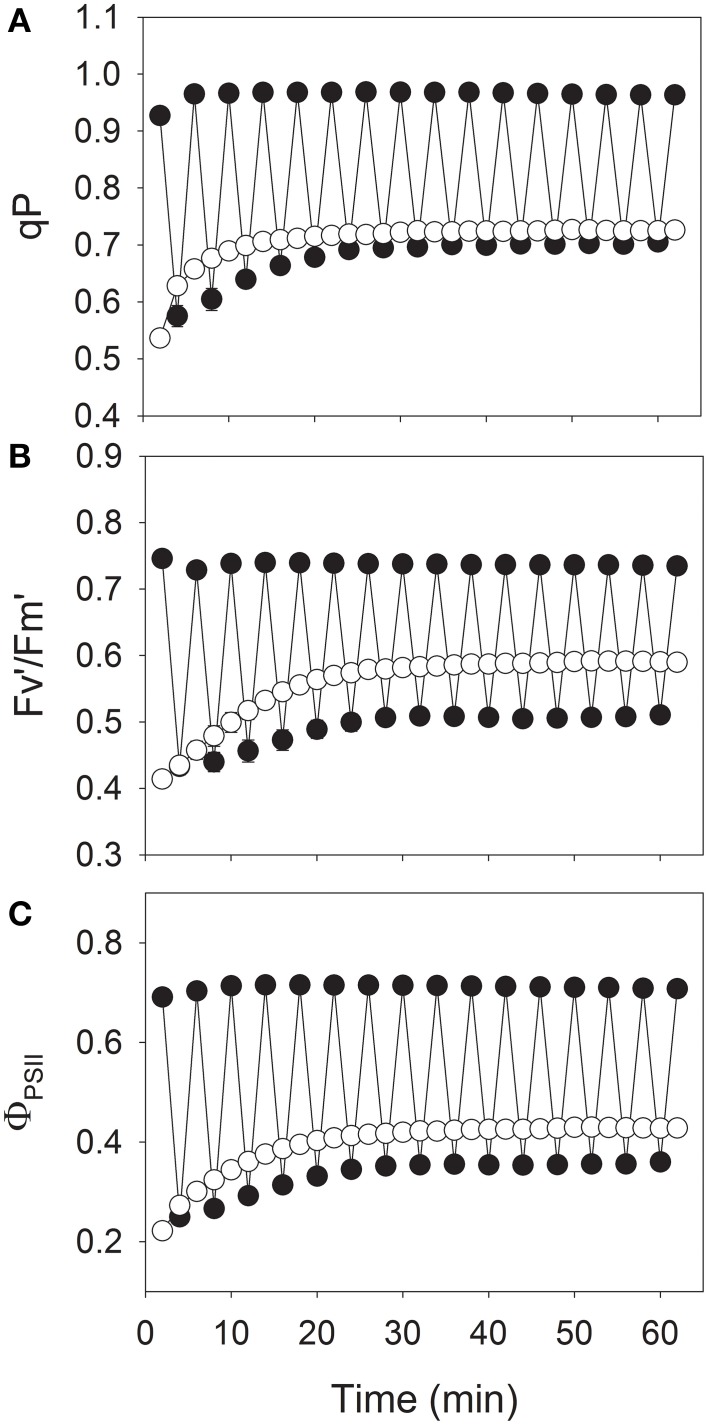
**Responses of coefficient of PSII photochemical quenching (qP) (A), maximum quantum yield of PSII after light-adaptation (*F*_*v*_′/*F*_*m*_′) (B), and effective quantum yield of PSII (Φ_PSII_) (C) to fluctuating light levels (closed symbols) or constant bright light (open symbols)**. Treatment protocol followed that described for Figure 2. Measurements were conducted at 25°C and 400 μmol mol^−1^ CO_2_. Values are means ± SE (*n* = 4).

By contrast, values for qP at 1200 μmol photons m^−2^ s^−1^ differed only slightly between the constant and fluctuating light treatments (Figure 3A), while *F*_*v*_′/*F*_*m*_′ and Φ_PSII_ at 1200 μmol photons m^−2^ s^−1^ were significantly lower under fluctuating light (*P* < 0.001; Figures 3B,C). The parameter *F*_*v*_′/*F*_*m*_′ represents the maximum efficiency of PSII when all reaction centers are “open,” and qP is the factor that relates maximum PSII efficiency to the operating PSII efficiency (Farage et al., 2006). Because Φ_PSII_ is the product of qP and *F*_*v*_′/*F*_*m*_′, the difference in Φ_PSII_ that we found between fluctuating light and constant light resulted from the change in *F*_*v*_′/*F*_*m*_′. These results suggested that although fluctuating light had little effect on the coefficient of PSII photochemical quenching, it induced a significant decline in the maximum efficiency of PSII.

**Figure 4:**
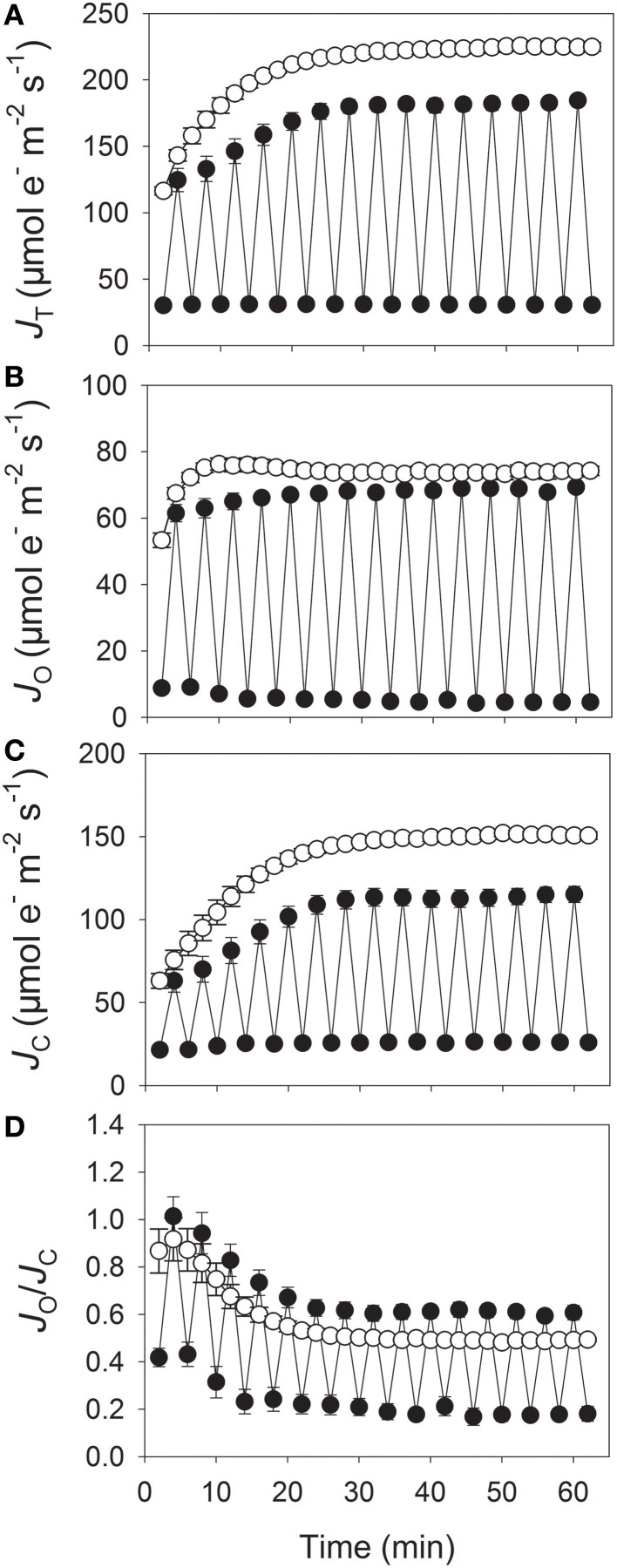
**Responses of total electron flow (*J*_T_) (A), electron flow devoted to RuBP carboxylation (*J*_C_) (B), electron flow devoted to RuBP oxygenation (*J*_O_) (C), and *J*_O_/*J*_C_ (D) to fluctuating light levels (closed symbols) or constant bright light (open symbols)**. Treatment protocol followed that described for Figure 2. Measurements were conducted at 25°C and 400 μmol mol^−1^ CO_2_. Values are means ± SE (*n* = 4).

After 60 min of treatment, total electron flow through PSII (*J*_T_) at 1200 μmol photons m^−2^ s^−1^ was significantly higher under constant illumination than under fluctuating light, i.e., 225 vs. 185 μmol electrons m^−2^ s^−1^, respectively (Figure 4A). During that time period, the value for electron flow devoted to RuBP oxygenation (*J*_O_) at 1200 μmol photons m^−2^ s^−1^ changed only slightly between constant- and fluctuating-light treatments (Figure 4B). By contrast, electron flow devoted to RuBP carboxylation (*J*_C_) at 1200 μmol photons m^−2^ s^−1^ was higher under constant light (151 μmol electrons m^−2^ s^−1^) than under fluctuating light (115 μmol electrons m^−2^ s^−1^) (Figure 4C). Consequently, the ratio *J*_O_/*J*_C_ at 1200 μmol photons m^−2^ s^−1^ was higher for plants treated with fluctuating light because of the lower value for *J*_C_ (Figure 4D). These results indicated that fluctuations in irradiance levels suppressed photosynthetic electron flow, primarily by restricting electron flow devoted to RuBP carboxylation. By comparison, electron flow devoted to RuBP oxygenation was hardly affected by fluctuating light conditions.

**Figure 5:**
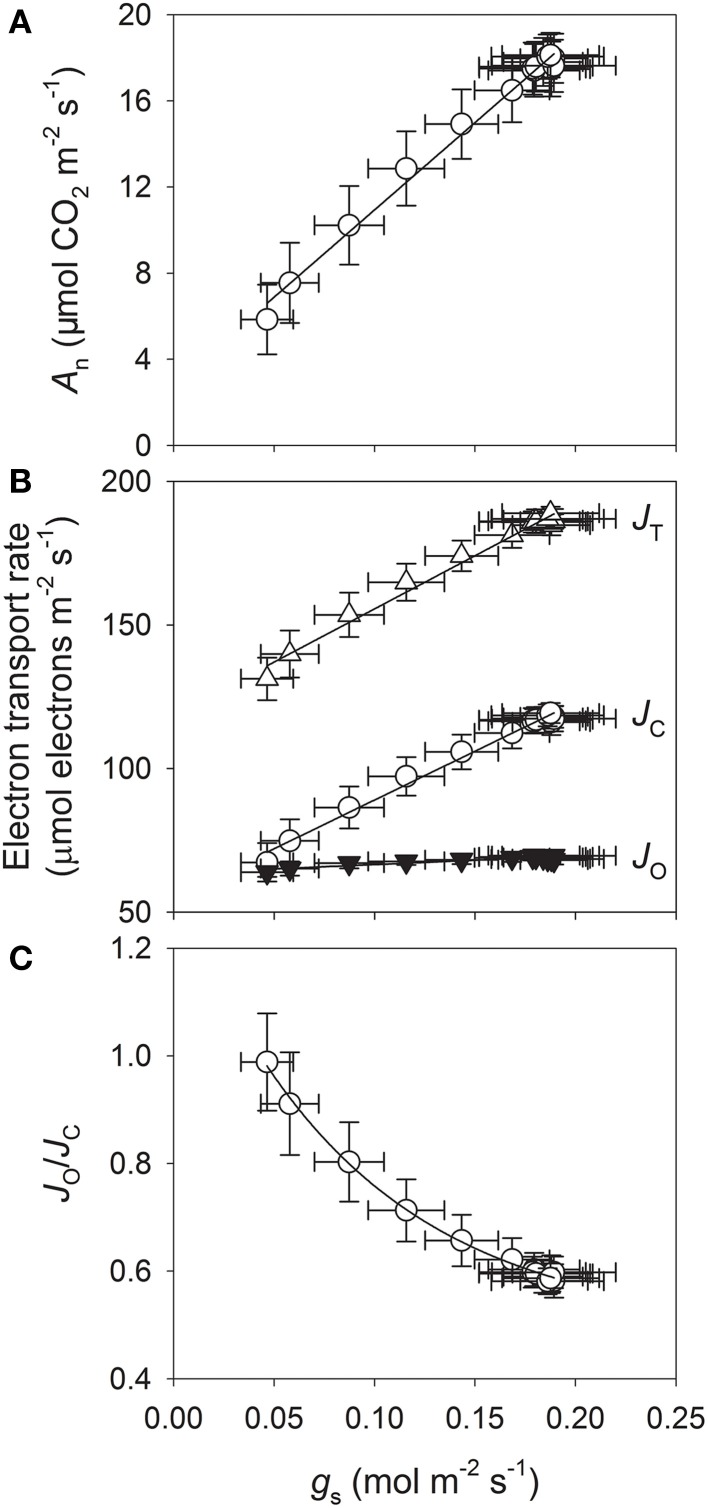
**Relationships between parameters derived from simultaneous measurements of gas exchange and chlorophyll fluorescence at 1200 μmol photons m^−2^ s^−1^ under fluctuating-light conditions**. Analyzed data are those depicted in Figures 2, 4. Comparisons were made between **(A)** *g*_s_ and *A*_n_; **(B)** *g*_s_ and *J*_T_, *J*_C_, or *J*_O_; and **(C)** *g*_s_ and *J*_O_/*J*_C_.

After pooling the photosynthesis data collected at 1200 μmol photons m^−2^ s^−1^ under fluctuating light, we determined that *g*_s_ was linearly and positively correlated with *A*_n_, *J*_T_, and *J*_C_ (Figures 5A,B). We found it interesting that *J*_O_ was independent of *g*_s_ (Figure 5B), which implied that RuBP carboxylation and RuBP oxygenation responded differently to *g*_s_. Under fluctuating light, *J*_O_ remained at nearly the maximum level throughout the experimental period. In the initial stage of fluctuating light treatment, electron flow attributed to RuBP oxygenation contributed largely to the total electron transport through PSII (Figure 5C).

**Figure 6:**
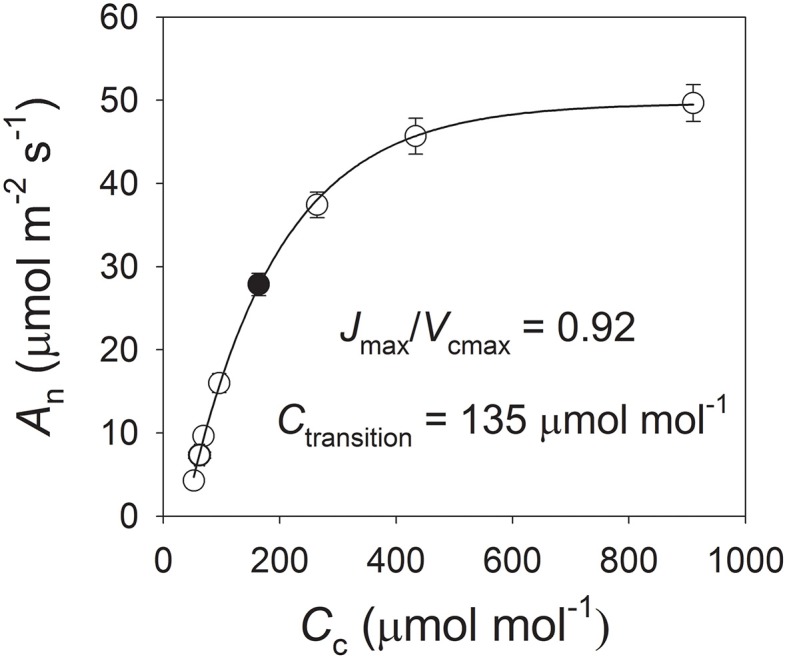
**Response of CO_2_ assimilation rate (*A*_n_) to incident chloroplast CO_2_ concentration (*C*_c_) at 25°C and 1200 μmol photons m^−2^ s^−1^**. Maximum rates of RuBP regeneration (*J*_max_) and RuBP carboxylation (*V*_cmax_) were calculated according to method of Long and Bernacchi (2003). Chloroplast CO_2_ concentration at which RuBP carboxylation transitions to RuBP regeneration (*C*_trans_), as determined by method of Yamori et al. (2010); Yamori et al. (2011). Solid symbol, *A*_n_ at atmospheric CO_2_ concentration of 400 μmol mol^−1^.

**Figure 7:**
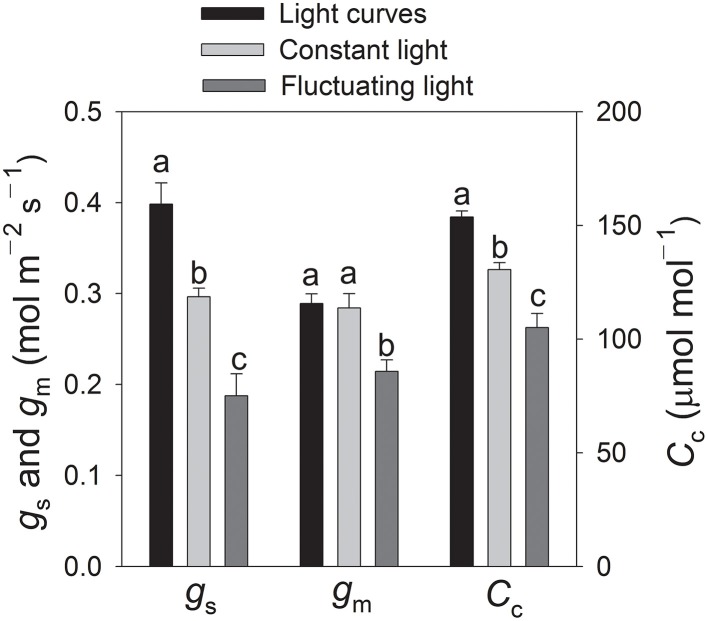
**Values for stomatal conductance (*g*_s_), mesophyll conductance (*g*_m_), and chloroplast CO_2_ concentration (*C*_c_) at 1200 μmol photons m^−2^ s^−1^ analyzed in light response curves, constant vs. fluctuating light levels after 60 min of treatment**. Measurements were conducted at 25°C and 400 μmol mol^−1^ CO_2_. Values are means ± SE (*n* = 4). For each treatment type, different letters indicate significant differences among light treatments (*P* < 0.05), based on Tukey's multiple comparison tests.

To analyze the limiting step of CO_2_ assimilation under fluctuating light, we examined the relationship between photosynthesis and chloroplast CO_2_ concentration. Here, the ratio of the maximum rate of RuBP regeneration (*J*_max_) to that of RuBP carboxylation (*V*_cmax_) was 0.92, and the chloroplast CO_2_ concentration at which the transition from RuBP carboxylation to RuBP regeneration occurred (*C*_trans_) was 135 μmol mol^−1^ (Figure 6). After exposure to fluctuating light conditions for 60 min, *g*_m_ at 1200 μmol photons m^−2^ s^−1^ was 0.21 mol m^−2^ s^−1^, which was significantly lower than that found with light curves (0.29 mol m^−2^ s^−1^) or under constant light (0.28 mol m^−2^ s^−1^) (Figure 7). For the light curves, *C*_c_ at 1200 μmol photons m^−2^ s^−1^ was 154 μmol mol^−1^. After exposure to fluctuating light or constant light for 60 min, the value for *C*_c_ was 105 or 131 μmol mol^−1^, respectively. This indicated that fluctuating light not only decreased *g*_s_ but also restricted *g*_m_, leading to a decline in *C*_c_. Because *C*_c_ was significantly lower than *C*_trans_ (*P* < 0.0001), the rate of CO_2_ assimilation at 1200 μmol photons m^−2^ s^−1^ under fluctuating light was limited by RuBP carboxylation.

## Discussion

The limiting step of *A*_n_ is mainly determined by the relative values of *C*_trans_ and *C*_c_. For tobacco plants supplied with high concentrations of nitrogen, photosynthesis tends to be limited by RuBP regeneration because *C*_c_ is higher than *C*_trans_ (Yamori et al., 2010, 2011). Such previous conclusions have been based on experiments that involved high levels of *g*_s_ and *g*_m_ under constant strong light. Although *g*_s_ and *A*_n_ can be significantly inhibited under fluctuating light, the limiting step of *A*_n_ under such conditions has been unknown. Here, our results indicate that both *g*_s_ and *g*_m_ are significantly restricted under fluctuating light, leading to the decrease in *C*_c_. After exposure to fluctuating light for 60 min, *C*_*c*_ was 105 vs. 135 μmol mol^−1^ for *C*_trans_. Those data provided evidence that, at 1200 μmol photons m^−2^ s^−1^, the photosynthetic process is limited by RuBP carboxylation under fluctuating light. Meanwhile, the high activation of photorespiration contributed largely to the regulation of photosynthetic electron flow.

Carriquí et al. (2015) have demonstrated that *g*_m_ plays an important role in determining the CO_2_ assimilation rate, especially at high light intensities. Nevertheless, the response of *g*_m_ to light intensity remains controversial. For example, in sclerophylls such as *Banksia integrifolia, B. serrata*, and *B. paludosa*, the average *g*_m_ under ambient CO_2_ concentration is 22% lower at 500 than at 1500 μmol photons m^−2^ s^−1^ (Hassiotou et al., 2009). However, the average *g*_m_ calculated for wheat leaves is not affected by light intensity (Tazoe et al., 2009). In tobacco leaves, *g*_m_ is significantly lower at 250 than at 1000 μmol photons m^−2^ s^−1^ (Flexas et al., 2007). By contrast, Yamori et al. (2010) have shown that *g*_m_ differs little between constant high light and constant low light in tobacco leaves. Our results indicated that *g*_m_ at 1200 μmol photons m^−2^ s^−1^ under the fluctuating light was 25% lower than the level calculated at constant light of 1200 μmol photons m^−2^ s^−1^ (Figure 7). We believe that this difference was caused by the use of a low light regime (100 μmol photons m^−2^ s^−1^) in fluctuating light. We found that, under fluctuating light, *g*_m_ was regulated by both high and low light levels; i.e., although the former induced an increase in *g*_m_, this effect could be partially reversed when plants were then exposed to reduced irradiance.

According to the photosynthesis model of Farquhar et al. (1980), CO_2_ assimilation in C_3_ plants is constrained by RuBP carboxylation and/or RuBP regeneration. Therefore, based on that model, the limiting step can be altered in two ways: (1) adjustments in the balance between the maximum rates of RuBP regeneration and RuBP carboxylation, or (2) changes in the chloroplast CO_2_ concentration (Hikosaka et al., 2006; Yamori et al., 2011). For example, in research with tobacco plants, Yamori et al. (2011) have reported that the CO_2_ assimilation rate at 380 μmol mol^−1^ CO_2_ and 1500 μmol photons m^−2^ s^−1^ (*A*_380_) depends upon the leaf-N content and is mainly determined by *J*_max_/*V*_cmax_. Furthermore, at high leaf-N content, *A*_380_ is limited by RuBP regeneration due to the low ratio of *J*_max_/*V*_cmax_ (Yamori et al., 2010, 2011). However, those conclusions have been drawn from experiments with plants that had high values for both *g*_s_ and *g*_m_, and which did not consider the effects of fluctuating light levels. By comparison, our photosynthetic data for *g*_s_, *A*_n_, *J*_T_, and *J*_max_/*V*_cmax_ ratio are very similar to those that describe the performance of plants grown with a high nitrogen supply (Yamori et al., 2011), indicating that plants grown with high N concentration were used in the present study. Furthermore, CO_2_ assimilation rate at 1200 μmol photons m^−2^ s^−1^ under constant light was limited by RuBP regeneration. When plants were exposed to fluctuating light, the declines in *g*_s_ and *g*_m_ resulted in a decrease in *C*_c_. The low light regimes under fluctuating light decreased the Rubisco activation state (Yamori et al., 2012), which further restricted the Calvin cycle. The rate of CO_2_ assimilation under high light during the fluctuating-light treatment tended to be limited by RuBP carboxylation. Fluctuating light has altered the limiting step of CO_2_ assimilation in tobacco plants with high leaf-N content.

Previous studies with *A. thaliana* have investigated the roles of cyclic electron flow (CEF) and O_2_-dependent alternative electron sinks in regulating photosynthetic electron flow under fluctuating light (Suorsa et al., 2012; Kono et al., 2014). It is believed that CEF is essential for proper acclimation of PSI to such light condition (Suorsa et al., 2012). However, the contribution of photorespiration to photodamage under fluctuating light is small in *Arabidopsis* leaves sampled from plants exposed to low light (Kono et al., 2014). In tobacco, the capacity of the photorespiratory pathway is strongly influenced by the growth light intensity, with sun leaves up-regulating this pathway to control CO_2_ assimilation and photosynthetic electron flow (Huang et al., 2014). However, it is unknown what role the photorespiratory pathway has in enabling plants normally grown under high light to adapt to fluctuating light conditions. Our data demonstrated that, when plants were exposed to fluctuating light, the reduction in *C*_c_ meant that less electron flow could be devoted to RuBP carboxylation. However, we found that the flow devoted to RuBP oxygenation was completely and highly activated under such conditions.

Suppression of CO_2_ fixation can cause over-acidification of lumen in the thylakoid membrane, which then activates non-photochemical quenching (NPQ) to dissipate excess light energy harmlessly as heat (Flexas and Medrano, 2002; Takahashi et al., 2007; Huang et al., 2012). At 1200 μmol photons m^−2^ s^−1^, *F*_*v*_′/*F*_*m*_′ were lower under fluctuating light than under constant light. Because *F*_*v*_′/*F*_*m*_′ is inversely related to NPQ, this result was evidence of the higher activation of NPQ under fluctuating light. Furthermore, an increase in the proton gradient across the thylakoid membrane can limit linear electron flow (LEF) via cytochrome b6/f (Tikkanen and Aro, 2014). Consumption of photochemical energy, such as ATP and NADPH, through the photorespiratory pathway is thought to alleviate such over-acidification. Especially in the initial stage of our fluctuating-light period, electron flow that was consumed by the photorespiratory pathway largely contributed to the operation of LEF. Therefore, for tobacco plants grown under full sunlight, photorespiratory pathway would be essential for regulating photosynthetic electron flow under fluctuating light, even though our findings contradict a previous report concerning low-light-grown *A. thaliana* (Kono et al., 2014).

Although photorespiratory intermediates such as glycine and glycerate inhibit the Calvin cycle (Chastain and Ogren, 1989; Eisenhut et al., 2007; Timm et al., 2012), they can be converted to glycerate-3-phosphate through the photorespiratory pathway (Peterhansel and Maurino, 2011). This process is critical for photosynthesis and photoprotection (Takahashi et al., 2007). Under fluctuating light, a reduction in *C*_c_ will accelerate RuBP oxygenation and, ultimately, the production of those intermediates. If the photorespiratory pathway is maintained at a low level under such conditions, the accumulation of those intermediates inhibits CO_2_ assimilation as well as photosynthetic electron flow, causing acceleration of photodamage (Chastain and Ogren, 1989; Eisenhut et al., 2007; Takahashi et al., 2007). In plants with a high rate of CO_2_ assimilation, rapid acceleration of photorespiratory pathway results in low glycine and glycerate contents (Timm et al., 2012). Therefore, to overcome those detrimental effects of photorespiratory intermediates, this pathway is highly activated under fluctuating light, which then benefits photosynthetic CO_2_ assimilation and photosynthetic electron flow. In addition, the operation of this pathway is necessary for the regeneration of RuBP (Takahashi et al., 2007). To optimize photosynthetic CO_2_ fixation, the rates of RuBP oxygenation and RuBP regeneration through photorespiratory pathway must be balanced. Therefore, under fluctuating light conditions, strong activation of the photorespiratory pathway accelerates RuBP regeneration, preventing a decrease in the RuBP pool and favoring the Calvin cycle.

In summary, our results provide evidence that, for sun-grown tobacco leaves, fluctuating light conditions significantly decrease both stomatal and mesophyll conductances, as well as chloroplast CO_2_ concentration. Consequently, the rate of CO_2_ assimilation is limited by RuBP carboxylation under such conditions. Meanwhile, the photorespiratory pathway is highly activated to regulate photosynthetic electron flow and benefit photosynthetic CO_2_ fixation. Thus, strong activation of this pathway is an important strategy by which sun-grown plants adapt to fluctuating light.

### Conflict of interest statement

The authors declare that the research was conducted in the absence of any commercial or financial relationships that could be construed as a potential conflict of interest.

## Abbreviations

*A*_n_: CO_2_ assimilation rate
*C*_c_: chloroplast CO_2_ concentration
*F_v_′*/*F_m_′*: the maximum quantum yield of PSII after light adaptation
*C*_trans_: the chloroplast CO_2_ concentration at which the transition from RuBP carboxylation to RuBP regeneration occurred
Φ_PSII_: effective quantum yield of PSII
*g*_m_: mesophyll conductance
*g*_s_: stomatal conductance
*J*_C_: electron flow devoted to RuBP carboxylation
*J*_O_: electron flow devoted to RuBP oxygenation
*J*_T_: total electron flow through PSII
*J*_max_: the maximum rate of RuBP regeneration
NPQ: non-photochemical quenching
PSI: photosystem I
PSII: photosystem II
qP: coefficient of PSII photochemical quenching
*V*_cmax_: the maximum rate of RuBP carboxylation.
